# The response of human macrophages to 3D printed titanium antibacterial implants does not affect the osteogenic differentiation of hMSCs

**DOI:** 10.3389/fbioe.2023.1176534

**Published:** 2023-06-21

**Authors:** Amaia Garmendia Urdalleta, Mathijs Van Poll, Niamh Fahy, Janneke Witte-Bouma, Willem Van Wamel, Iulian Apachitei, Amir A. Zadpoor, Lidy E. Fratila-Apachitei, Eric Farrell

**Affiliations:** ^1^ Department of Biomechanical Engineering, Faculty of Mechanical, Maritime and Materials Engineering, TU Delft, Delft, Netherlands; ^2^ Department of Oral and Maxillofacial Surgery, Erasmus MC, University Medical Center Rotterdam, Rotterdam, Netherlands; ^3^ Department of Orthopaedics, Erasmus MC, University Medical Center Rotterdam, Rotterdam, Netherlands; ^4^ Department of Applied Science, Technological University of the Shannon: Midlands Midwest, Limerick, Ireland; ^5^ Department of Medical Microbiology and Infectious Diseases, Erasmus MC, University Medical Center Rotterdam, Rotterdam, Netherlands

**Keywords:** osteoimmunomodulation, titanium bone implants, human macrophages, human marrow stromal cells, silver nanoparticles

## Abstract

Macrophage responses following the implantation of orthopaedic implants are essential for successful implant integration in the body, partly through intimate crosstalk with human marrow stromal cells (hMSCs) in the process of new bone formation. Additive manufacturing (AM) and plasma electrolytic oxidation (PEO) in the presence of silver nanoparticles (AgNPs) are promising techniques to achieve multifunctional titanium implants. Their osteoimmunomodulatory properties are, however, not yet fully investigated. Here, we studied the effects of implants with AgNPs on human macrophages and the crosstalk between hMSCs and human macrophages when co-cultured *in vitro* with biofunctionalised AM Ti6Al4V implants. A concentration of 0.3 g/L AgNPs in the PEO electrolyte was found to be optimal for both macrophage viability and inhibition of bacteria growth. These specimens also caused a decrease of the macrophage tissue repair related factor C-C Motif Chemokine Ligand 18 (CCL18). Nevertheless, co-cultured hMSCs could osteogenically differentiate without any adverse effects caused by the presence of macrophages that were previously exposed to the PEO (±AgNPs) surfaces. Further evaluation of these promising implants in a bony *in vivo* environment with and without infection is highly recommended to prove their potential for clinical use.

## 1 Introduction

Despite the major efforts and advances in the field of orthopaedic implants, several complications including implant-associated infections (IAIs) and aseptic loosening persist (Dutch Athroplasty Register, 2019), causing a huge burden to millions of patients (Tedesco et al., 2017). The development of biomaterials that can support the integration of the implants in the body while also providing multiple biofunctionalities without creating adverse effects is, therefore, an important current research area (Agrawal et al., 2013).

In recent years, researchers have begun to investigate the role of the immune system in bone homeostasis following implantation (Franz et al., 2011; Lee et al., 2019; Liu and Segura, 2020; Negrescu and Cimpean, 2021). It is well known that biomaterial implantation in the body is followed by monocyte recruitment to the wound site and differentiation into macrophages (Franz et al., 2011; Chen et al., 2016). These macrophages play a crucial role in determining the outcome of the biomaterial integration depending on their response towards the surface. They can either enhance tissue repair including guiding the new bone formation process in and around the implant or create an inflammatory response that results in fibrous tissue encapsulation and implant failure (Miron and Bosshardt, 2016). In very general terms, macrophage behaviour ranges between two fundamental states: classically activated/M1 macrophages which secrete pro-inflammatory cytokines and play a key role in the early stage of inflammation by eliminating external pathogens and tissue debris, and alternatively activated/M2 macrophages which are characterized by the secretion of anti-inflammatory cytokines while enhancing tissue repair (Davenport Huyer et al., 2020). Both polarization states are necessary for bone tissue regeneration, but it is only when a fine balance is maintained that macrophages can release osteogenesis-enhancing factors and recruit MSCs, leading to a successful new bone formation (Mosser and Edwards, 2008; Fearing and Van Dyke, 2014; Chen et al., 2016).

The evidence of macrophage sensitivity to environmental cues (Sica et al., 2014) demands an adequate adjustment of the surface properties of biomaterials. This includes chemical composition, wettability, or topography, which can result in a desired macrophage activation pattern and subsequent osteogenesis, *i.e.,* osteoimmunomodulatory function (Lee et al., 2019; Chen et al., 2021). Currently, new implant surface designs are being actively developed and pursued to improve the clinical outcomes of implants and enhance their osteoimmunomodulatory properties (Amengual-Peñafiel et al., 2019; Lee et al., 2019; Negrescu and Cimpean, 2021).

Additive manufacturing (AM = 3D printing) has shown promising results in the development of novel metallic orthopaedic implants (van Hengel et al., 2020b; 2020a; Fazel et al., 2021). This technology enables the fabrication of metallic implants with easily tuneable and controllable shapes and microarchitectures to fit the defect area or generate personalized implants. The porous 3D printed structures provide large surface areas (Yuan et al., 2019) favourable for the adhesion of bone forming cells and the osteogenic differentiation of marrow stromal cells (MSCs) (Ahmadi et al., 2015; Zadpoor, 2015; Taniguchi et al., 2016). Furthermore, multiple biofunctionalities can be achieved through the incorporation of bioactives in the porous structure and/or surface physical and chemical modifications. In particular, biofunctionalisation via plasma electrolytic oxidation (PEO) has been shown to improve the biocompatibility of AM implants, allowing higher proliferation (van Hengel et al., 2017), osteogenic differentiation (Santos-Coquillat et al., 2019), and enhanced pro-repair ability of macrophages (Razzi et al., 2020).

Nevertheless, the increased surface area of AM implants represents also a niche for bacterial cells, increasing the risk of peri-implant infections which can subsequently lead to complications and implant failure (van Hengel et al., 2020b). Therefore, further biofunctionalisation of such implants through the incorporation of antibacterial elements is a vital step forward for the creation of suitable metallic AM orthopaedic implants. Extensive research has been performed to combine antibacterial and osteogenic functionalities within an implant, showing that antibacterial coatings incorporating silver (van Hengel et al., 2020a), strontium (Zhou et al., 2019), a combination of both silver and strontium (Geng et al., 2017) or silicon and copper (Shen et al., 2020) have the ability to eliminate the IAIs caused by bacteria such as *S. aureus (S. aureus)* or *E. coli (E. coli)* without causing cytotoxicity against osteogenic cells or hindering the normal osteogenesis process. However, more recent literature has revealed that these antibacterial agents may compromise the survival of immune cells, highlighting the need for further research in this field (Croes et al., 2018; Razzi et al., 2020).

The majority of the *in vitro* models developed for the study of the effects of modified titanium surfaces on monocytes/macrophages (Hotchkiss et al., 2016; Hamlet et al., 2019) and stem cells (Ingrassia et al., 2017; van Hengel et al., 2017; Kado et al., 2019) are focused on studying the behaviour of 1 cell type at a time (*i.e.,* monoculture models). By comparison, the studies investigating the effects of titanium modifications on the interactions between stem cells and immune cells (*i.e.,* co-culture models) are very scarce (Huang et al., 2019; Bai et al., 2020) and practically non-existent for modified AM porous titanium implants containing antibacterial elements. In this work, the effects of human macrophages on human marrow stromal cells (hMSCs) were studied for the first time when indirectly co-cultured in the presence of AM porous Ti6Al4V implants which were surface biofunctionalised by PEO in the presence of silver nanoparticles (AgNPs).

## 2 Materials and methods

### 2.1 Titanium implants fabrication by AM

Porous Ti6Al4V implants were fabricated following the design rationale and protocol previously described in our studies (van Hengel et al., 2017). Briefly, implants were fabricated using a selective laser melting (SLM) printer (SLM-125, Realizer, Borchem, Germany) that operated with a YLM-400-AC Ytterbium fiber laser (IPG Photonics Corporation, Oxford, United States) under an atmosphere containing argon and limited (<0.2%) oxygen content. A laser spot size of 145 µm and a layer thickness of 50 µm were used. The exposure time, wavelength, and laser power were 300 µs, 1,070 ± 10 nm, and 96 W, respectively. Medical grade (grade 23, ELI) Ti6Al4V powder particles (APC, Boisbriand, Quebec, Canada), which were spherical and with particle sizes of 10–45 µm were used. Following fabrication, the samples were vacuum cleaned and then ultrasonicated in acetone, 96% ethanol, and demineralised water to remove any possible loose particles that were created during the 3D printing process. The final samples had a length of 40 mm, a diameter of 0.5 mm and the pore size was between 300–400 µm.

### 2.2 Surface biofunctionalisation

The surface of the 3D printed implants was modified by PEO in electrolytes containing various concentrations of AgNPs (*i.e.,* 0 g/L, 0.15 g/L, 0.3 g/L, 0.75 g/L, 1.5 g/L and 3.0 g/L) resulting in six experimental groups (*i.e*., PEO, PEO + 0.15 Ag, PEO + 0.3 Ag, PEO + 0.75 Ag, PEO + 1.5 Ag and PEO + 3.0 Ag). Biofunctionalisation was performed by using a PEO research unit which included an AC power supply (50 Hz, type ACS 1500, ET Power Systems Ltd., Eyam, United Kingdom), a data acquisition board (SCXI, National Instruments, Austin, Texas, United States), a computer interface, a thermostatic bath (Thermo Haake V15, Karlsrhue, Germany), and an electrolytic cell made of double-walled glass, which contained 800 mL electrolyte. The AM Ti6Al4V implants represented the anode while a stainless-steel cylinder was used as the cathode. The PEO electrolyte contained 24.0 g/L calcium acetate and 4.2 g/L calcium glycerophosphate. In the series produced with electrolytes containing AgNPs, the nanoparticles (7–25 nm in size purchased from Sigma-Aldrich, St. Louis, United States) were added to the electrolyte in the specified concentrations and the mixture was sonicated twice for 5 min and stirred in between for 5 min to achieve uniform particles dispersion in the solution. The experimental conditions are summarised in Supplementary Table S1 (see the online Supplementary Material). Samples were oxidised under a current density of 20 A/dm^2^ for 5 min. The electrolyte was continuously stirred to maintain a homogeneous dispersion of particles in the electrolyte during the oxidation process. In addition, the electrolyte temperature was maintained at 7°C ± 1°C. The voltage transients (*V*-*t* curves) were recorded at a sampling rate of 1 Hz during the entire process. After PEO, the samples were cleaned under tap water for 1 min and air dried.

### 2.3 Implant characterisation

#### 2.3.1 SEM imaging

The morphology of the biofunctionalised implant surfaces was analysed using a scanning electron microscope (SEM, JSM-IT100LV, JEOL, Tokyo, Japan) with an electron beam energy ranging between 10–20 kV and 10 mm of working distance. Before imaging, the specimens were sputtered with a gold layer for 30 s to improve their electrical conductivity under SEM. Energy-dispersive X-ray spectroscopy (EDS) was used to analyse the elemental composition of the surfaces.

#### 2.3.2 Release of Ag ions

The release profiles of Ag ions were obtained by Inductively Coupled Plasma - Optical Emission Spectrometry (ICP-OES) using a Thermo Fisher iCAP6300 Duo instrument (Thermo Fisher Scientific, Waltham, Massachusetts, United States). Therefore, each implant was cut into pieces of 1.0 cm length, inserted into dark Eppendorf tubes (Eppendorf, Kerkenbos, Netherlands) with 1.0 mL of phosphate buffer saline (PBS), and incubated at 37°C (*n* = 3). The medium was collected and refreshed at every selected timepoint (*i.e.,* 2°h, 6 h, 12 h, 24 h, 2 d, 4 d, 7 d, 14 d, and 28 d) and the concentration of Ag was measured.

### 2.4 Cell isolation, seeding and culture on implant surfaces

#### 2.4.1 Human CD14^+^ monocyte isolation

The Sanquin Blood bank (Sanquin blood bank, Amsterdam, Netherlands; contract number: NVT0053.01) provided the buffy coats for this study after ethical approval. The buffy coats were transferred to a T175 flask (Flacon, St. Louis, United States) and were diluted with wash buffer containing PBS (Gibco, ThermoFisher Scientific, Waltham, Massachusetts, United States) supplemented with 0.1% w/v BSA (Sigma Aldrich, St. Louis, Missouri, United States) to a final volume of 240 mL. After an initial thrombocyte removal, 30 mL of the diluted blood was added to 50 mL tubes previously filled with 15 mL Ficoll (Ficoll-PaqueTM PLUS, GE Healthcare, Little Chalfont, UK). Density gradient separation was performed by spinning the tubes at 1000 *g* with no break for 15 min. Human peripheral blood mononuclear cells (hPBMCs) were obtained following the removal of the Ficoll/plasma interphase layer. CD14^+^ monocyte isolation was performed by first labelling hPBMCs with 100 µL of anti-CD14^+^ magnetic bead solution and subsequently applying the cell suspension to a CD14^+^ magnetic-activated cell sorting (MACS) column, according to manufacturer’s instructions (all materials from Miltenyi Biotec, Bergisch Gladbach, Germany).

#### 2.4.2 Human paediatric MSCs isolation and preculture

The isolation of human paediatric marrow stromal cells was performed using leftover iliac crest bone chip from 3 different male donors undergoing cleft palate reconstructive surgery (ages 9-10), as previously described (Knuth et al., 2018). The human material was harvested with the consent of the institution for the use of surgical waste material with a possibility for parental opt-out and the approval of the Erasmus Medical Centre Ethics Committee (MEC-2014-106). Paediatric hMSC were previously characterised by confirmation of multilineage differentiation capacity (Knuth et al., 2018).The cells were thawed and subsequently plated at 2,300 cells/cm^2^ in complete hMSC expansion medium (αMEM, Gibco, ThermoFisher Scientific, Breda, Netherlands) supplemented with 10% v/v heat inactivated foetal bovine serum (FBS) (Sigma Aldrich, St. Louis, Missouri, United States, lot #BCCD0778), 50 μg/mL gentamycin, 1.5 μg/mL Amphotericin B, 25 μg/mL L-ascorbic acid 2-phosphate (Sigma Aldrich, St. Louis, Missouri, United States), and 1 ng/mL fibroblast growth factor-2 (FGF-2) (Instruchemie, Delfzijl, Netherlands). The cells were then expanded in a T175 flask (Corning, Glendale, Arizona, United States) at 37°C and 5% CO_2_ in a humidified atmosphere until reaching 80% confluency.

#### 2.4.3 Cell seeding on implant surfaces

Prior to cell seeding, the implants were cut into pieces of 1.0 cm length and steam sterilised at 121°C for 21 min by using an autoclave. Under sterile conditions, each implant was placed in a 0.2 mL tubes (BIOplastics, Landgraaf, Netherlands) and seeded with 5 × 10^5^ human CD14^+^ monocytes in 100 µL of the X-vivo 15 medium (Lonza Group GA, Basel, Switzerland) supplemented with 20% v/v heat inactivated FBS, 50 μg/mL gentamycin, and 1.5 μg/mL Amphotericin B. For the case of hMSCs seeding, the trypsinised cells were seeded at a density of 1.5 × 10^5^ hMSCs in 100 µL of the complete MSC expansion medium. In both cases, the implants were incubated at 37°C for 2 h, while rotating the tubes 180° every 30 min to ensure that the cells adhered evenly to the whole implant surface area. After seeding, the samples with CD14^+^ monocytes were transferred to a 48-well plate with fresh 400 µL of X-vivo medium, while the samples with hMSCs were transferred to a non-treated 24 well plate (ThermoFisher Scientific, Denmark) with 500 µL of fresh complete hMSC expansion medium. The incubation times and the specific assays performed in monocultures are described in Sections 2.5, 2.7–2.9.

#### 2.4.4 Indirect co-culture of hMSCs and human macrophages

Firstly, hMSCs were isolated and seeded on various samples (as explained in Section 2.4.3.) and were incubated for 24 h in an expansion medium while monocytes were seeded on various samples (as explained in Section 2.4.3) and were incubated in the X-vivo medium for 48 h. After that time, both types of seeded implants were placed together in a non-treated 12 well plate containing 800 µL of co-culturing medium composed of high-glucose DMEM (Gibco, ThermoFisher Scientific, Breda, Netherlands) supplemented with heat-inactivated 10% v/v FBS, 50 μg/mL gentamycin, 1.5 μg/mL Amphotericin B, and 0.1 mM L-ascorbic acid 2-phosphate (Sigma-Aldrich, St. Louis, Missouri, United States). For each cell type, three seeded implants were placed in every well and a separating wall was formed with 0.5 mL of 2% w/v cell culture tested agarose with low gelling temperature (Sigma-Aldrich, St. Louis, Missouri, United States) in the middle of the well to avoid any possible direct contact between the two types of the cells. Both of them, however, shared the same medium. After 3 days of co-culture, the implants with hMSCs and macrophages were separated for further immunological and osteogenic evaluation, as described in Sections 2.7–2.9. Figure 7A shows the steps involved in the setting up of the co-culture. The co-culture experiments were performed three times.

### 2.5 Cell viability

The viability of the human macrophages incubated on implant surfaces was evaluated using a live/dead Viability/Cytotoxicity assay (Gibco ThermoFisher Scientific, Breda, the Netherlands) after 3 days of culture and for 2 different donors. Therefore, after 3 days of culture, the implants with the attached cells were rinsed three times in 0.9% w/v NaCl and subsequently incubated with 300 μL of 0.9% w/v NaCl solution containing 0.1% of Calcein AM and 0.15% of ethidium homodimer (EthD-1) for 40 min at 37°C. The implants were subsequently washed three times in a 0.9% w/v NaCl solution and were imaged with a fluorescent microscope (Zeiss Axiovert 200M, Breda, Netherlands) at a wavelength of 495/515 nm for Calcein AM and 495/635 nm for EthD-1.

### 2.6 Antimicrobial activity assay

Non-treated (NT), PEO, PEO + 0.3 Ag, and PEO + 3.0 Ag implants were placed inside calWells (Symcel, Spånga, Sweden). Per calWell, a total of 10^4^ colony forming units (CFUs) of *S. aureus* (ATCC 29213) or *E. coli* (ATCC 25922) were cultured in 200 μL of the X-vivo medium supplemented with 20% FBS. The experiment was performed in duplicate. After preparing the wells, they were placed in a calScreener (Symcel, Spånga, Sweden) to perform isothermal microcalorimetry, through which the heat produced by the metabolic activity of the bacteria was determined in real time for a period of 24 h. After assessing the metabolic responses of the bacteria in the presence of the implants containing different concentrations of AgNPs, the implants were prepared for SEM imaging. Towards that aim, they were fixed with 4% w/v paraformaldehyde (PFA) and 1% v/v glutaraldehyde in PBS for 2 hours at 4°C. Then, the samples were dehydrated in gradually increasing ethanol concentrations (50, 70%, and 96%), were incubated in the presence of hexamethyldisilazane (HDMS) for 20 min, and were dried in an Eppendorf tube (Eppendorf, Hamburg, Germany) for at least 2 hours. Finally, the samples were coated with a thin gold layer and were imaged by SEM (SEM, JSM-IT100LV, JEOL, Tokyo, Japan) using an electron beam energy of 10 kV and a working distance of 10 mm.

### 2.7 Calcium concentration in the culture medium

Three implants with adhered hMSCs were transferred to a well of a 24 well-plate containing 250 µL of an osteogenic induction medium with the following composition: high-glucose DMEM (Gibco, ThermoFisher Scientific, Breda, Netherlands) supplemented with 10% v/v heat inactivated FBS, 50 μg/mL gentamycin, 1.5 μg/mL Amphotericin B, fresh 0.1 µM dexamethasone, 0.1 mM L-ascorbic acid 2-phosphate, and 10 mM β-glycerophosphate (Sigma-Aldrich, St. Louis, Missouri, United States). Technical triplicates were prepared for each experimental group. The cells were cultured at 37°C and 5% CO_2_ and the medium was refreshed every 3-4 days. The implants were moved to another well plate at day 14 to avoid the interference of the cells detached from the implants. The controls included implants with no cells. At days 3, 7, 10, 14, 17, 21, and 24, 200 µL of the cell culture supernatant was collected and stored at −20°C. An eight-point standard curve was prepared using CaCl_2_ at a concentration range of 0–3 mM in calcium-free αMEM (Gibco, ThermoFisher Scientific, Breda, Netherlands, catalogue n. # 041-91867M, lot #2283388) and CaCl₂ standard values were used to calculate the calcium concentration present in each sample. 10 μL of sample was mixed with 100 µL of a calcium reagent (1 + 1 mix of 1 M ethanolamine pH 10.5 and 0.35 mM o-cresolphthalein complexone, 19.8 mM 8-hydroxyquinoline and 0.6 M hydrochloric acid, all from Sigma-Aldrich, St. Louis, Missouri, United States) and the optical density of each sample was determined by a VersaMax spectrophotometer (Molecular Devices, San Jose, California, United States) at a wavelength of 570 nm. The same procedure was applied for the co-cultured hMSCs after 3 days of indirect co-culture with human macrophages. The hMSC-containing implants were further cultured for 21 days in the osteogenic medium. Samples of the culture supernatant were collected at timepoints 3, 7, 10, 14, 17, and 21 days and the calcium assay was performed.

### 2.8 Protein secretion analysis

An enzyme-linked immunosorbent assay (ELISA) was used to study the factors secreted by the human macrophages when cultured on the implant surfaces. Commercially available ELISA DuoSet Development Kits (R&D Systems, McKinley, Minneapolis, United States) were used to measure the secretion levels of the pro-inflammatory cytokine IL-6 and tissue-repair related chemokine CCL18 present in the supernatant. Therefore, the collected medium at day 3 for 3 different donors was centrifuged for 5 min at 500 *g* and were stored at −80°C until the assay was performed according to the manufacturer’s instructions. An ELISA assay was also performed for the macrophages with co-cultured the hMSCs: after 3 days of indirect co-culture, the macrophages were transferred to a non-adherent 24 well plate with 500 µL of the co-culturing medium. After 24 h, 400 µL of the cell supernatant was collected and stored at −80°C. The protein secretion levels of the (co-cultured) macrophages were normalized to the DNA content of the macrophages attached to each implant. Therefore, the implants with adhered macrophages were harvested at the same time with the medium collected for ELISA and were stored at −80°C. The DNA quantification was performed using a CYQUANT cell proliferation assay (Invitrogen, Carlsbad, California, United States) following the manufacturer’s instructions.

### 2.9 Gene expression analysis

The human macrophages monocultured on implant surfaces were harvested at day 3, were lysed by the addition of 400 µL of the TRizol reagent (Thermo Fisher Scientific, Waltham, United States), and were stored at −80°C. The co-cultured macrophages were harvested after 3 days of indirect co-culture and were lysed through the addition of 400 µL of RNA STAT-60 (Tel-Test, Friendswood, Texas, US). While the hMSCs monocultured on the implant surfaces were harvested at day 7, the co-cultured hMSCs were harvested after 3 days of co-culture and another 7 days of monoculture. All the hMSC specimens were lysed through the addition of 400 µL of RNA STAT-60. For RNA isolation, a phase separation step was applied in which 80 µL of chloroform was added to each sample followed by centrifugation for 15 min at 12.000 *g*. The RNA content was extracted from the aqueous phase, mixed with an equal volume of 70% v/v ethanol, and loaded into a RNeasy micro-column (Qiagen, Germantown, United States). The isolation was performed following the manufacturer’s protocol. The total isolated RNA was quantified by means of a spectrophotometer/fluorometer (DSS-11 Series Spectrophotometer/fluorometer, DeNovix, Wilmington, United States) at 260/280 nm. For cDNA synthesis, 0.15 μg of RNA was used per human macrophage sample, 0.75 μg for monocultured hMSCs, and 0.6 μg for co-cultured hMSCs. This procedure was performed according to the instructions of the manufacturer of the RevertAid First Strand cDNA kit (Thermo Fisher Scientific, Waltham, Massachusetts, United States). Gene expression quantification was performed by a qPCR analysis where 5.0 µL of 2x qPCR mastermix [TaqMan Universal PCR mastermix (Thermo Fisher Scientific, Waltham, Massachusetts, United States)], or qPCR Mastermix Plus for SYBR GreenI (Eurogentec, Seraing, Belgium), 0.5 µL of primer mix, and 2.5 µL of ddH₂O were mixed with 2 µL of cDNA. The signal of each sample was measured by means of a Bio-Rad CFX96 Real-Time PCR Detection system (Bio-Rad, Hercules, California, United States). The list of macrophage-specific and osteogenic-specific genes used for the qPCR analysis can be found in Table 1. The primers and probes of each gene are listed in more detail in Supplementary Tables S2–S4, (Supplementary Material). The best housekeeper index (BKI) was calculated by performing the geometric mean expression of the genes Glyceraldehyde-3-phosphate (*GAPDH*), Beta-2-Microglobulin (*B2M*), and Ubiquitin C (*UBC*). Subsequently, the gene expression of each sample relative to the BKI expression was calculated using the ΔΔCt method where Gene Expression = $2^{-∆Cq}$ and *ΔCq* = *CqSample—CqBKI*. This assay was performed for 3 different donors for each cell type.

**TABLE 1:** The immune-specific and osteogenic-specific genes used for the qPCR analysis.

IMMUNE FACTORS (for macrophages)						OSTEOGENIC FACTORS (for hMSCs)
Pro-inflammatory cytokines	Anti-inflammatory			Tissue-repair chemokines	Pro-osteogenic cytokines	
	Cytokines	Cell surface markers	Growth factors			
*TNF*					*OSM*	*COL1*
*IL6*	*IL10*	*CD163*	*TGFB1*	*CCL18*	*PTGS2*	*RUNX2*
*IL1B*	*IL1RA*	*MCR1*			*BMP2*	*ALPL*
						*IBSP*

### 2.10 Statistical analysis

IBM SPSS 25.0 was employed for the statistical evaluation in this study. The figures present the mean values ± standard deviation. The Kolmogorov-Smirnov test was used to assess the normality condition of the data. Then, a linear mixed model was used followed by the Bonferroni *post hoc* test. The different implant conditions were considered as fixed factors and the donors as random factors.

## 3 Results

### 3.1 Surface biofunctionalisation of AM Ti6Al4V implants by PEO

**FIGURE 1:**
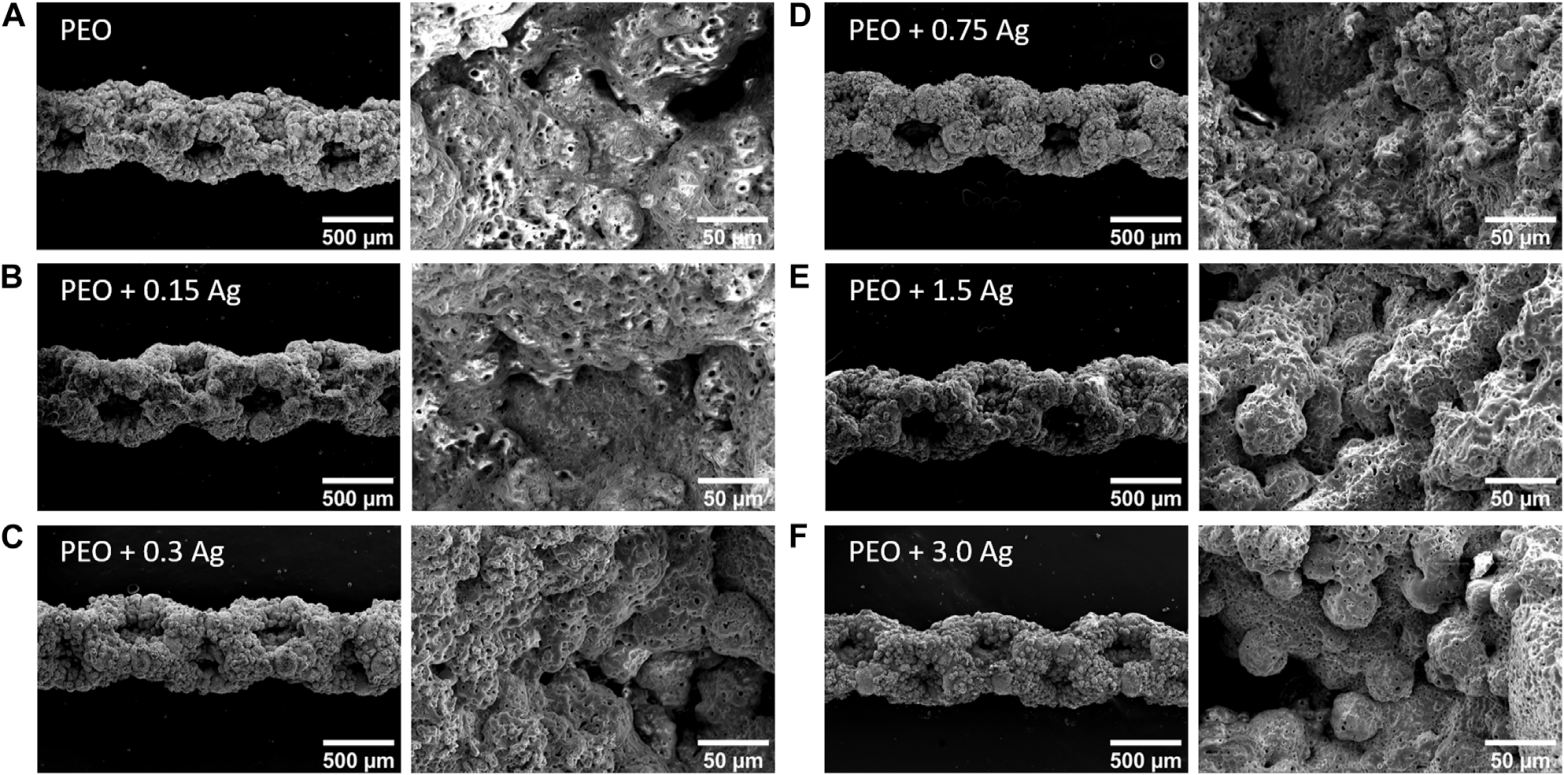
PEO biofunctionalisation creates characteristic micro- and nano-porous surfaces on the AM Ti6Al4V implants. The SEM images of the PEO-treated **(A)** and PEO + 0.15 g/L **(B)**, 0.3 g/L **(C)**, 0.75 g/L **(D)**, 1.5 g/L **(E)**, and 3 g/L **(F)** AgNPs implants.

**FIGURE 2:**
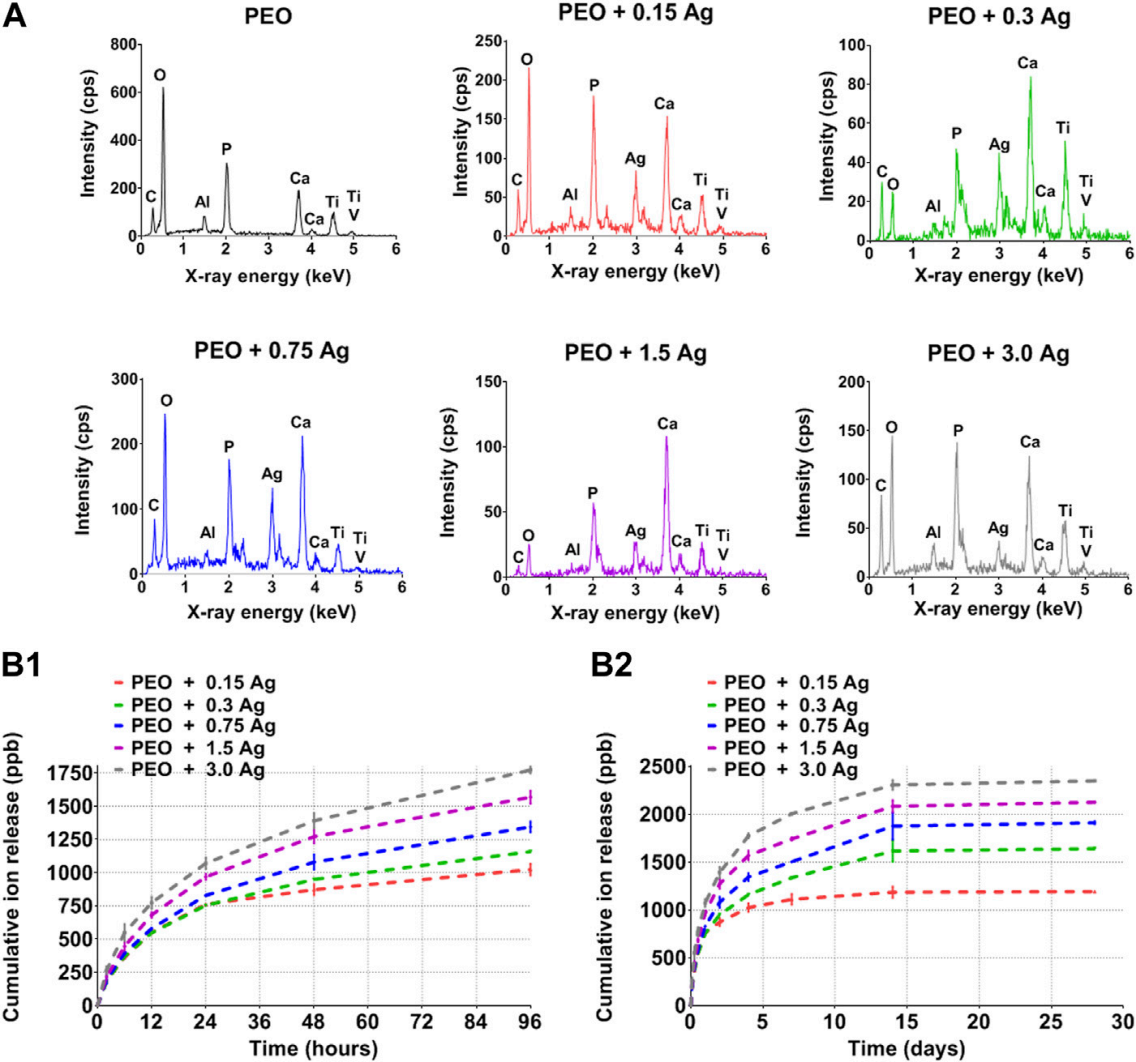
The PEO biofunctionalisation and incorporation of AgNPs at different concentrations form distinct surface chemical compositions and Ag ion release profiles. **(A)** The EDS spectra of PEO-treated and PEO + 0.15 g/L, 0.3 g/L, 0.75 g/L, 1.5 g/L, and 3 g/L AgNPs. **(B1, B2)** The cumulative release of Ag ions from the PEO + AgNPs implants (0.15 g/L, 0.3 g/L, 0.75 g/L, 1.5 g/L and 3.0 g/L) as measured by ICP-OES after 2 h, 6 h, 12 h, 24 h, 2 d, 4 d, 7 d, 14 d, and 28 d of immersion in PBS. Data represents the mean ± standard deviation (*n* = 3).

The PEO treatment of the AM implants modified the surface morphology through the creation of a porous titanium oxide layer with interconnected pores up to few μm in diameter (Figure 1A). The incorporation of AgNPs in the PEO layers did not change the morphology of the surfaces (Figures 1B–F), as observed by SEM imaging, and also reflected in the comparable voltage transients for all the implants (Supplementary Figure S1). The EDS analysis revealed the main elements of the metallic implants, namely, titanium (Ti), aluminium (Al), vanadium (V) together with oxygen (O), calcium (Ca), and phosphorus (P) incorporated during the PEO process (Figure 2A). In the case of the surfaces with AgNPs, the incorporation of the nanoparticles was confirmed by the presence of Ag peaks in the EDS spectra. Since Ag ions (Ag^+^) leaching from AgNPs is known to be one of the mechanisms contributing to the antimicrobial and cytotoxic effects (Beer et al., 2012; Albers et al., 2013; McShan et al., 2014; Qing et al., 2018), the Ag ion release profiles were also assessed (Figure 2B). All the implants revealed a high release rate in the first 4 days of their immersion with concentrations ranging from 1,038 ppb Ag^+^ for the lowest AgNPs concentration to 1855 ppb Ag^+^ for the highest (Figure 2B1). Between days 4 and 14, the release rate decreased for all the surfaces, although less so for the PEO + 0.3 Ag, PEO + 0.75 Ag, PEO + 1.5 Ag, and PEO + 3.0 Ag implants, which showed a 50% increase in the cumulative concentrations of Ag^+^. After 14 days, the cumulative concentrations of Ag^+^ stabilised until the end of the assay, indicating minimal release of Ag^+^ (Figure 2B2).

### 3.2 Effects of AgNPs incorporated in the PEO layers on human macrophages and bacterial cells

**FIGURE 3:**
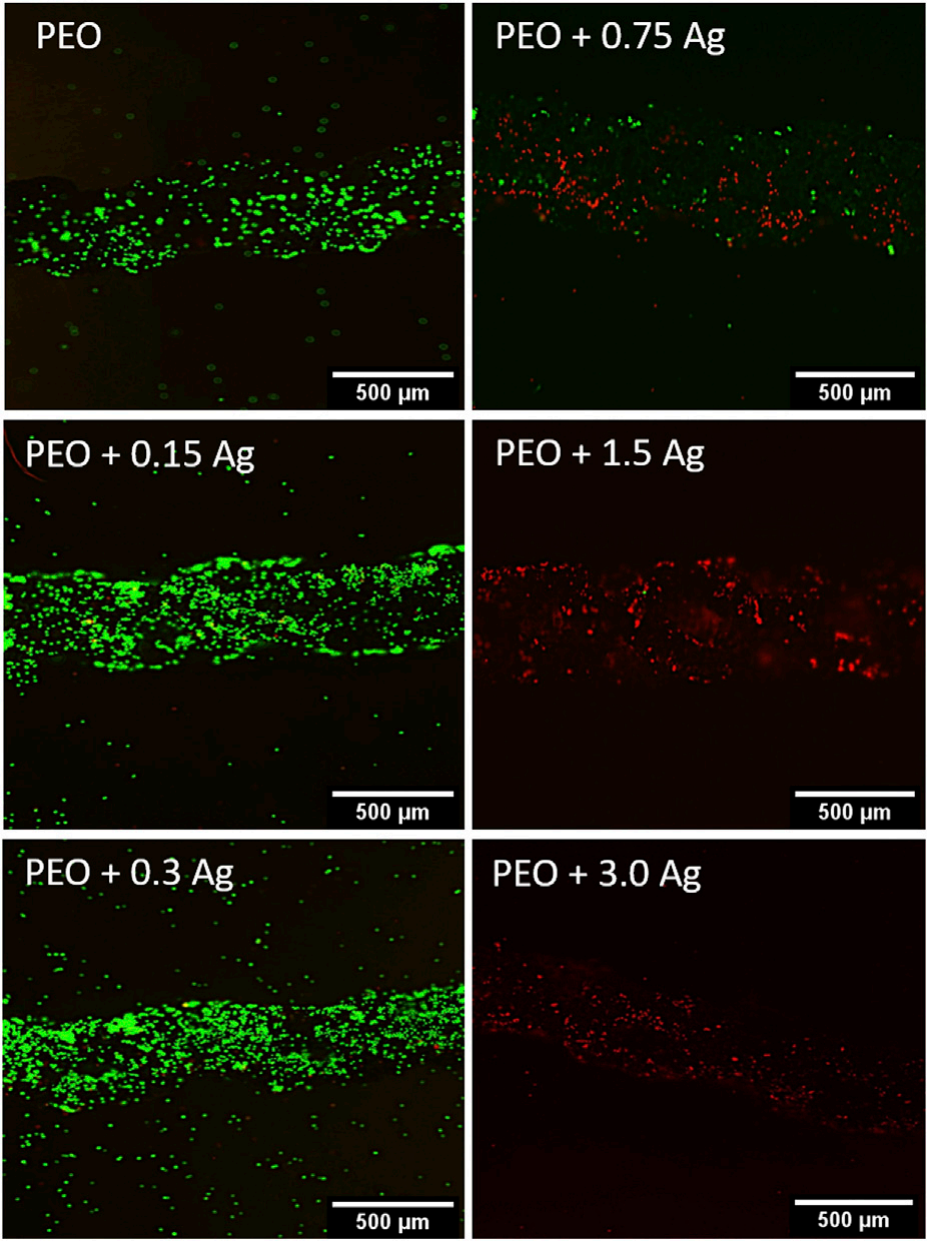
PEO + 0.3 Ag implants support the viability of primary human macrophages. The live/dead staining of human macrophages on the PEO, PEO + 0.15, PEO + 0.3, PEO + 0.75, PEO + 1.5, and PEO + 3.0 implants after 96 h of culture. The green and red colours indicate viable and dead cells, respectively (*N* = 2 donors and *n* = 3 implants per condition).

The cytotoxic effect of the implants containing different concentrations of AgNPs on the human macrophages was assessed by culturing cells from two different donors on the surfaces of the implants for 4 days. The cells were able to adhere to all the implants but, as indicated by the live/dead staining, only the PEO, PEO + 0.15Ag, and PEO + 0.3Ag implants proved to be non-toxic against human macrophages (Figure 3). The PEO-modified implants with 0.75 g/L AgNPs or higher showed cytotoxic effects on the human macrophages.

**FIGURE 4:**
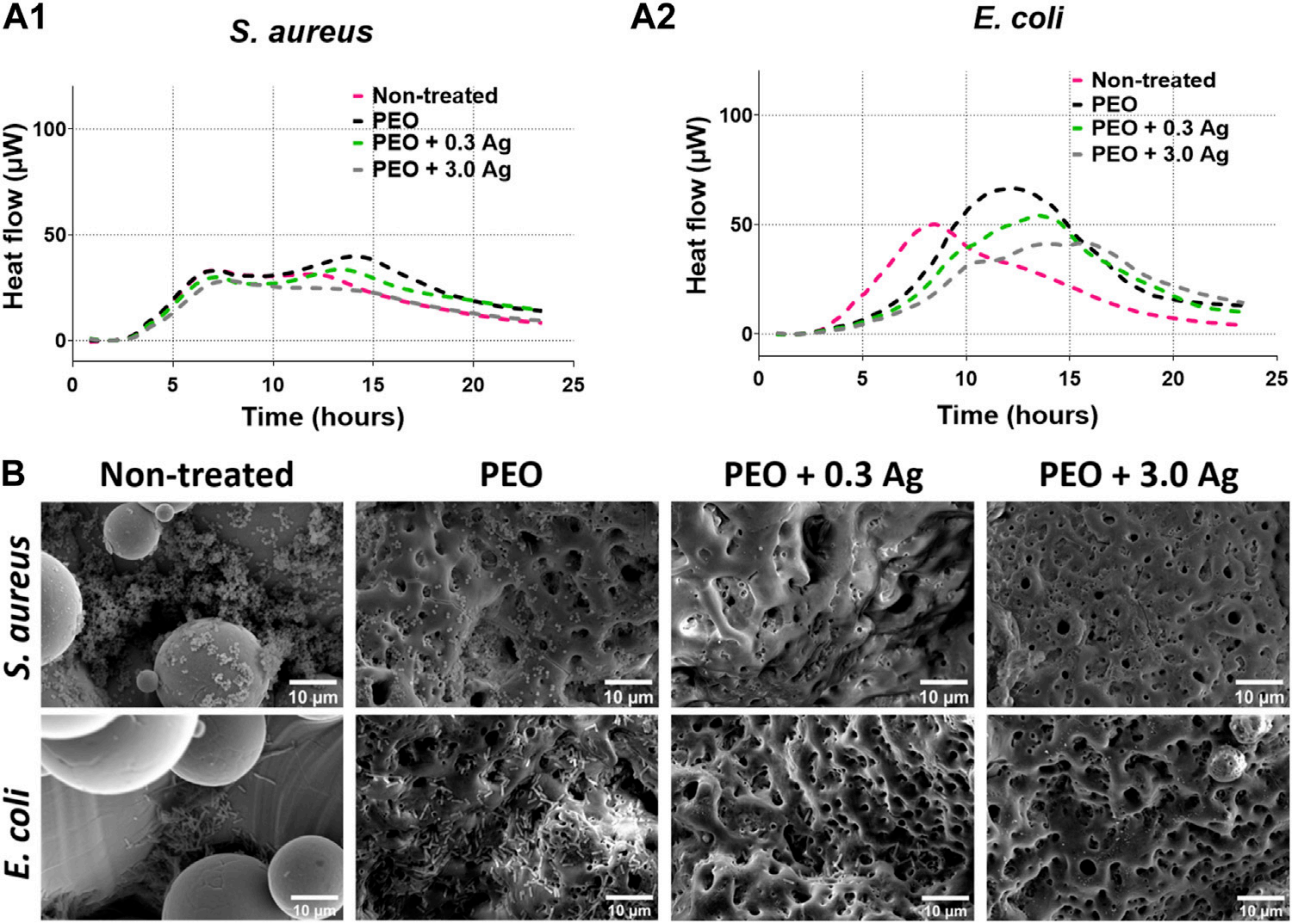
The PEO + 0.3 Ag implants reduce metabolic activity and biofilm formation by *E. coli* and *S. aureus*. The metabolic activity of **(A1)** *S. aureus* and **(A2)** *E. coli* in the presence of the non-treated, PEO, PEO + 0.3 Ag, and PEO + 3.0 Ag implants as assessed by microcalorimetry over 24 h. **(B)** The SEM images of *E. coli* and *S. aureus* biofilm formation on non-treated, PEO, PEO + 0.3 Ag, and PEO + 3.0 Ag implants after 24 h in the presence of 10^4^ CFU. The experiment was performed 3 times in duplicate.

To investigate the antibacterial activity of the implants containing the different concentrations of AgNPs, specifically NT, PEO, PEO + 0.3 Ag, and PEO + 3.0 Ag, the metabolic response of *S. aureus* and *E. coli* was investigated using microcalorimetry. The effects of all the implants on the metabolic activity of *S. aureus* was minimal (Figure 4A1). Nevertheless, more pronounced effects were observed in the case of *E. coli* (Figure 4A2). While the *E. coli* cultured in the presence of the NT samples showed an increase in the metabolic activity already after 3 h, the *E. coli* incubated with the other groups showed a delayed increase in the metabolic activity at later times (after ca. 6 h). In addition, the peak in the metabolic activity decreased when the *E. coli* was incubated in the presence of the PEO implants incorporating AgNPs. The largest decrease was observed in the presence of the PEO + 3.0 Ag implants.

Importantly, SEM imaging (Figure 4B) revealed that both *S. aureus* and *E. coli* were able to colonise the NT surfaces to a high degree. By comparison, when biofunctionalised by PEO, less bacteria were present on the surfaces, although *E. coli* was still able to spread out and colonize the implants. Furthermore, no *S. aureus* and very few *E. coli* were visible on the PEO surfaces with AgNPs indicating that these implants exhibited antibacterial activity.

In summary, the incorporation of AgNPs at a concentration of 0.3 g/L could reduce bacteria attachment on implant surfaces while being non-toxic for human macrophages.

### 3.3 Effects of PEO treated implants on the polarisation of human macrophages

**FIGURE 5:**
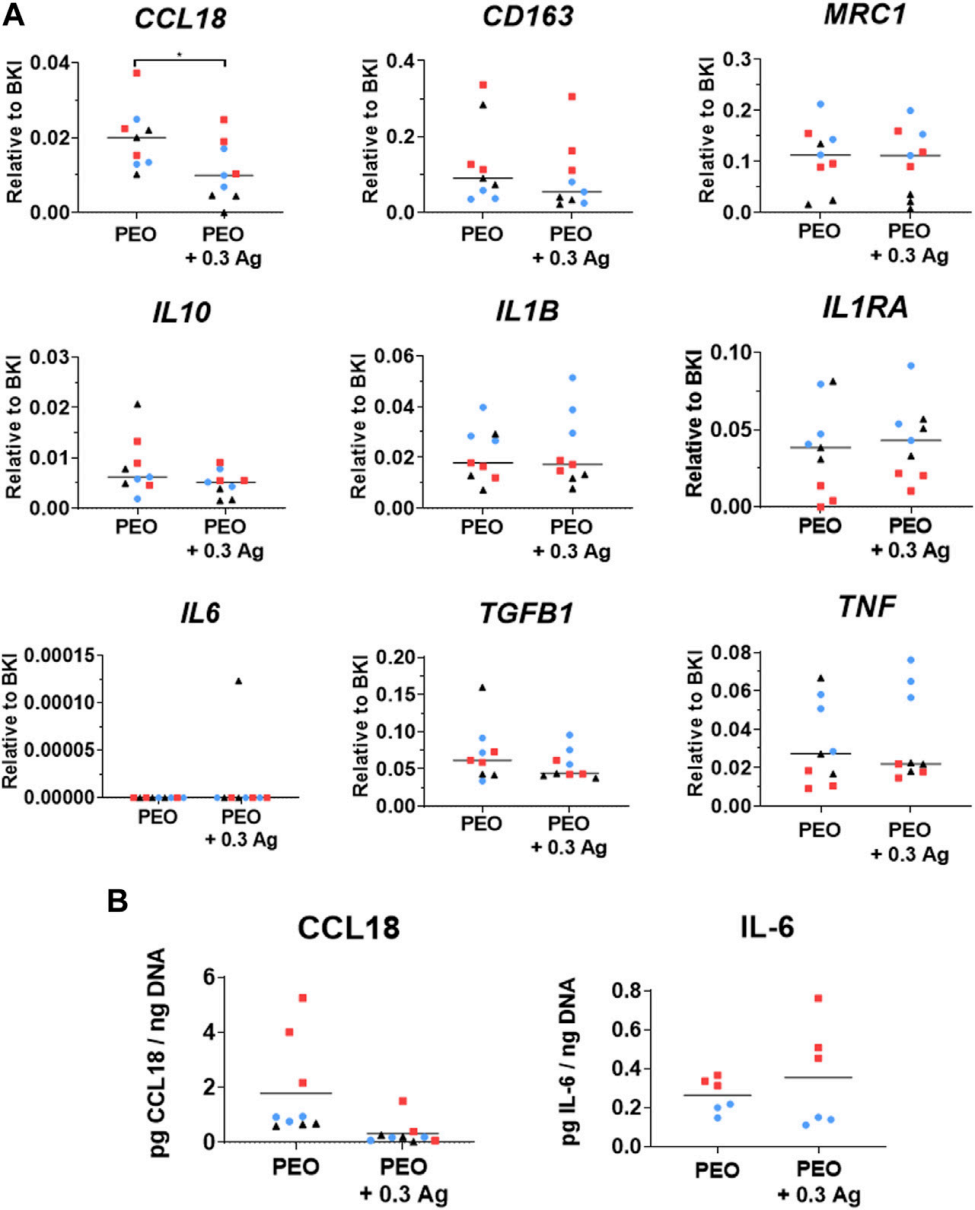
The PEO and PEO + 0.3 Ag implants similarly modify the behaviour of primary human macrophages, as revealed by the markers expressed at gene and protein levels. **(A)** The gene expression levels of inflammatory markers following 4 days of culture of human macrophages on the PEO or PEO + 0.3 Ag implants. *p* ≤ 0.05 (*). **(B)** The CCL18 and IL-6 protein levels present in the cell culture supernatant of primary human macrophages cultured on the PEO or PEO + 0.3 Ag implants for 4 days, with protein concentration normalised to DNA. (*N* = 3 donors; *n* = 3). Each donor is represented by a different geometrical symbol and colour.

The PEO + 0.3 g/L AgNPs implants were further investigated regarding their effects on the macrophage behaviour. The PEO only group was used as a control. In general, the nine genes indicative of macrophage response/polarisation revealed comparable expression levels on the PEO and PEO + 0.3 Ag implants after 4 days of culture for 3 different donors (Figure 5A). No significant differences were observed between both types of implants for the pro-inflammatory cytokines interleukin 1 Beta (*IL1B*) and tumor necrosis factor (*TNF*), while interleukin 6 (*IL6*) was not detected at all in any of the groups. The trend was the same for the anti-inflammatory factors interleukin 1 receptor antagonist (*IL1RA*), interleukin 10 (*IL10*), cluster of differentiation 163 (*CD163*), and mannose Receptor C-Type 1 (*MRC1*). The only significant gene expression difference between the implant types was observed for the tissue-repair chemokine *CCL18* with a small but significantly higher expression (*p* < 0.05) detected on the PEO specimen. In a similar manner, the normalised ELISA measurements of the proteins secreted by the human macrophages cultured on the implant surfaces for 4 days indicated similar secretion levels of the pro-inflammatory cytokine IL-6 and tissue-repair chemokine CCL18 on both implant types (Figure 5B).

### 3.4 Effects of PEO treated implants on hMSCs osteogenic differentiation

**FIGURE 6:**
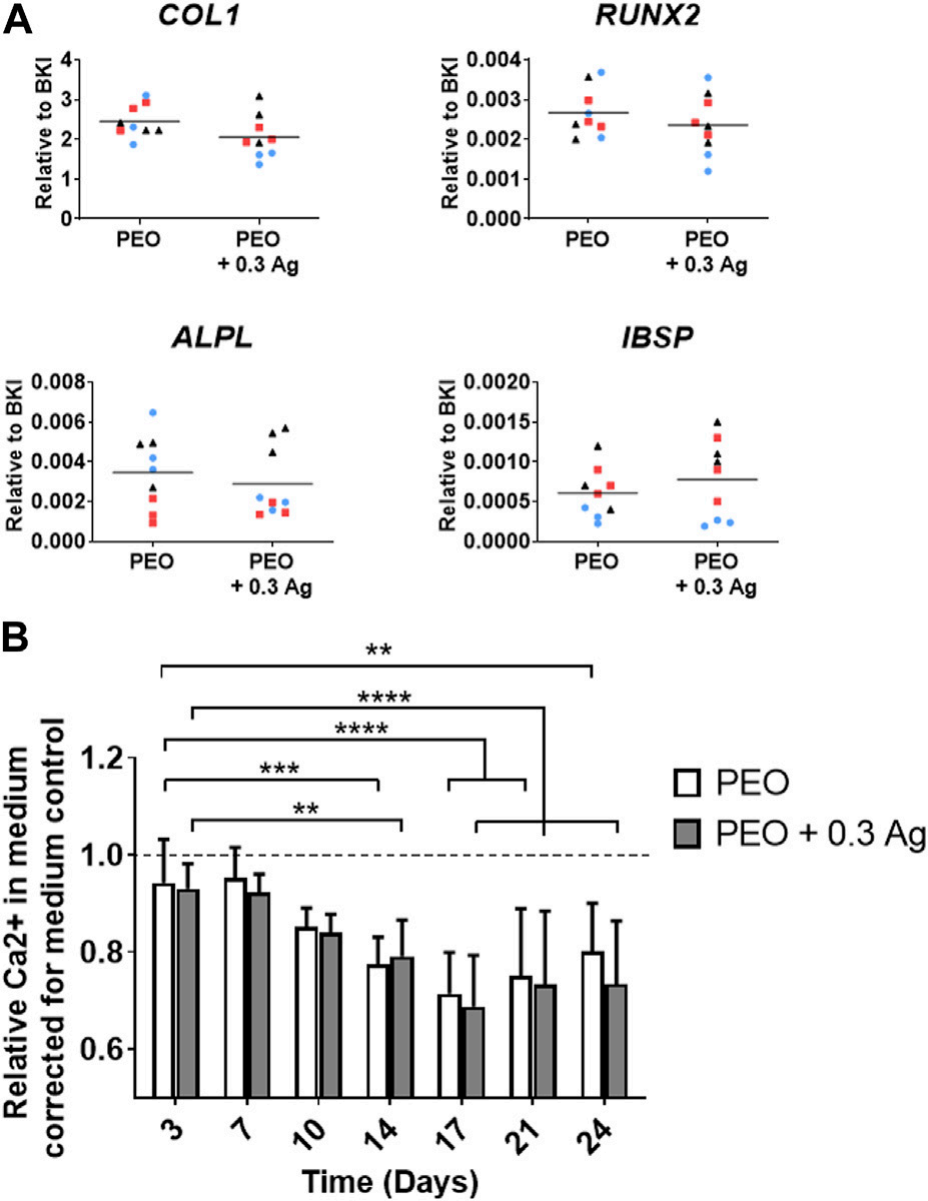
PEO + 0.3 Ag implants support osteogenesis and mineralisation by hMSCs. **(A)** The gene expression levels of osteogenesis-related genes in hMSCs cultured on the PEO and PEO + 0.3 Ag implants after 7 days of culture. **(B)** The relative Ca^2+^ concentration in the culture medium for the hMSCs cultured on the PEO and PEO + 0.3 Ag implants over a period of 24 days. *p* ≤ 0.01 (**), *p* ≤ 0.001 (***), *p* ≤ 0.0001 (****). (*N* = 3 donors; *n* = 3). Each donor is represented by a different geometrical symbol and colour.

To assess the osteogenesis of hMSCs when cultured on the implants, the expression of selected genes related to osteogenesis [collagen type 1 (*COL1*), runt-related transcription factor 2 (*RUNX2*), alkaline phosphatase (*ALPL*) and integrin binding sialoprotein (*IBSP*)] was analysed for 3 different donors (Figure 6A). The results showed comparable levels for the cells cultured on both PEO and PEO + 0.3 Ag implants at day 7 of the culture (Figure 6A). The process of osteogenic differentiation of hMSCs on both implant types was also monitored by measuring the calcium concentration in the medium over time. The hMSCs cultured osteogenically on the implant surfaces significantly increased their calcium uptake from day 14 onwards, as evidenced by a decrease in the concentration of Ca^2+^ in the culture medium (Figure 6B) which is indicative of the mineralisation of the extracellular matrix on the implant surface. As in the case of gene expression, no significant differences were observed in the calcium uptake between the hMSCs cultured on the PEO and PEO + 0.3 Ag implants.

Taken together, these findings suggest that hMSCs could osteogenically differentiate and mineralise the extracellular matrix when seeded on both PEO and PEO + 0.3 Ag implants.

### 3.5 Effects of the PEO treated implants on the co-culture of macrophages and hMSCs

**FIGURE 7:**
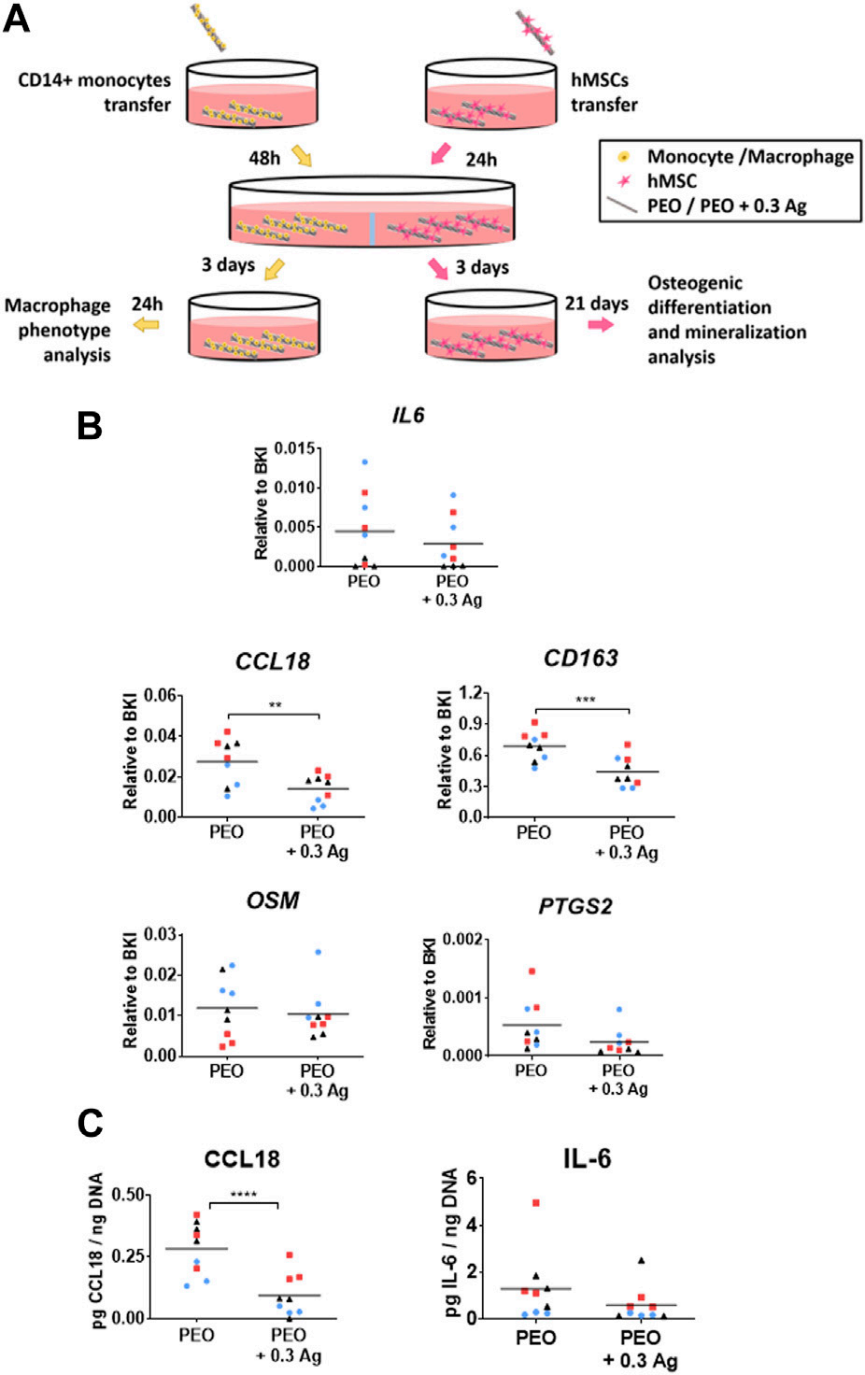
Primary human macrophages co-cultured with hMSCs on the PEO + 0.3 Ag implants polarize as on the PEO-only treated surfaces but possess slightly less pro-repair potential. **(A)** A schematic diagram of the indirect co-culture setup. **(B)** The gene expression levels of immune factors following 3 days of co-culture of human macrophages on the PEO or PEO + 0.3 Ag implants with hMSCs. *p* ≤ 0.01 (**), *p* ≤ 0.001 (***). **(C)** The CCL18 and IL-6 protein levels present in the cell culture supernatant of primary human macrophages co-cultured with hMSCs on the PEO or PEO + 0.3 Ag implants for 3 days in co-culture and 1 day in monoculture, with protein concentration normalised to DNA. *p* ≤ 0.0001 (****). (*N* = 3 donors; *n* = 3). Each donor is represented by a different geometrical symbol and colour.

To investigate the effects of the PEO + 0.3 Ag implants on the human macrophage response and how that cellular response affects the osteogenic differentiation of hMSCs, an indirect co-culture model was developed in this study (Figure 7A).

*IL6* was similarly expressed by the macrophages cultured on both specimens after 3 days of co-culture (Figure 7B) whereas the tissue-repair related gene *CCL18* was upregulated on the PEO surfaces (*p* ≤ 0.01) as compared to the PEO + 0.3 Ag implants. Similarly, the levels of the surface marker *CD163* expressed predominantly by anti-inflammatory macrophages were significantly higher on the PEO-treated implants (*p* ≤ 0.001). In the case of the pro-osteogenic genes expressed by macrophages [oncostatin M (*OSM*), prostaglandin-endoperoxide synthase 2 (*PTGS2*), bone morphogenetic protein 2 (*BMP2*)], two out of three genes were detectable in both implant types after 3 days of co-culture. This trend was followed by all the three donors. Comparable expression levels of *OSM* and *PTGS2* were measured on the PEO and PEO + 0.3 Ag implants.

The ELISA measurement of the proteins secreted by the co-cultured macrophages and normalised to the DNA content (Figure 7C) revealed that the PEO treated implants induced a higher secretion of CCL18 per cell (*p* ≤ 0.0001) as compared to the PEO + 0.3 Ag implants. On the contrary, the normalised IL-6 levels were comparable between PEO and PEO + 0.3 Ag. This follows the same trend as for the gene expression results.

**FIGURE 8:**
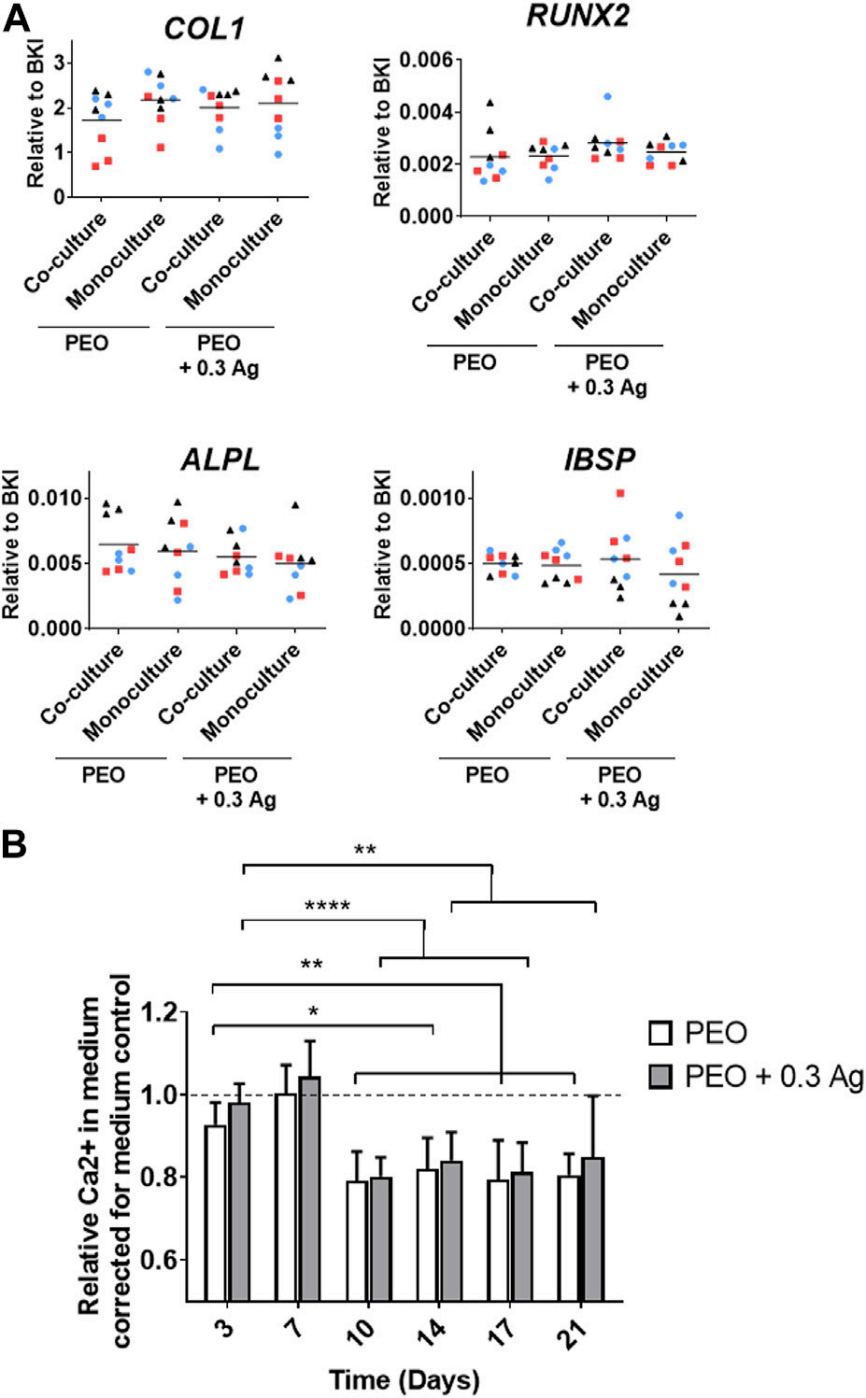
Mono- or co-cultured hMSCs with primary human macrophages on implant surfaces show similar osteogenic behaviour. **(A)** The gene expression levels of osteogenesis-related genes in hMSCs cultured on the implant surfaces in mono- or co-culture after 7 days in osteogenic medium. **(B)** The relative Ca^2+^ concentration in the culture medium for the hMSCs cultured on the implant surfaces over a period of 21 days. *p* ≤ 0.05 (*), *p* ≤ 0.01 (**), *p* ≤ 0.0001 (****). (*N* = 3 donors; *n* = 3). Each donor is represented by a different geometrical symbol and colour.

The osteogenic differentiation ability of the co-cultured hMSCs was assessed by analysing the expression of several osteogenesis-promoting genes (*COL1, RUNX2, ALPL,* and *IBSP*). These factors were similarly expressed by the hMSCs cultured on both PEO and PEO + 0.3 Ag implants after 3 days of co-culture with macrophages followed by 7 days of monoculture in the osteogenic medium. In addition, no gene expression differences were observed between the mono- or co-cultured hMSCs on neither type of specimens (Figure 8A). This tendency was consistent for all the donors.

The osteogenic differentiation of the co-cultured hMSCs was further confirmed by analysing the calcium concentration in the medium when cultured in monoculture for 21 days (following the 3 days of co-culture). A significant drop in the Ca^2+^ concentration in the culture medium was observed from day 10 onwards on both PEO and PEO + 0.3 Ag surfaces (Figure 8B). No differences were observed between both types of implants.

Summarising, when co-cultured with hMSCs, the macrophages generated a more pro-repair environment on the PEO surfaces than on PEO + 0.3 Ag. The same immune response was observed for the monoculture, as explained in Section 3.3. In comparison, no differences in the pro-inflammatory and pro-osteogenic responses were observed between both groups after 3 days of co-culture. On the other hand, these results indicate that the hMSCs cultured in both mono- and co-culture and in the presence or absence of AgNPs could osteogenically differentiate.

## 4 Discussion

Further improvements in the clinical success of bone implants call for multifunctional biomaterials and advanced fabrication technologies to minimise or prevent adverse effects, such as peri-implant infections, maximise osseointegration, and address more patient-related factors (*e.g.,* through personalised implants). Understanding the interplay between the different types of cells at the implant-tissue interface is essential for the development of implants that can fulfil the above clinical needs. In this study, the interactions of hMSC and human primary macrophage were studied for the first time when co-cultured on AM porous titanium implants modified by PEO with AgNPs. Our findings revealed that the incorporation of properly-dosed AgNPs in the PEO neither adversely affects macrophages nor alters the osteogenic behaviour of hMSCs, while endowing the implants with an antibacterial behaviour.

The incorporation of AgNPs by PEO on the surface of biomedical titanium alloys provides an antibacterial functionality, as demonstrated so far *in vitro* and *ex vivo* (van Hengel et al., 2017; 2020b; 2020c; Van Hengel et al., 2020c). Although the PEO layers produced with 3.0 g/L AgNPs showed no cytotoxic effects for preosteoblasts (van Hengel et al., 2020b) and hMSCs (van Hengel et al., 2017; Razzi et al., 2020), a more recent study indicated that these implants may be cytotoxic against human macrophages (Razzi et al., 2020). In this study, we observed a dose-dependent cytotoxicity behaviour: out of the five different AgNPs concentrations varying between 0.15 and 3.0 g/L AgNPs, the human macrophages were only viable on the PEO + 0.15 Ag and PEO + 0.3 Ag implants (after 4 days). Combining these results with the cumulative ion release profiles of these implants suggests that human macrophages could endure 1,181 ppb of Ag ions, the concentration measured for PEO + 0.3 Ag implants at day 4. This level of Ag ions was also observed to not affect the osteogenic differentiation of hMSCs. Even higher levels of Ag could be tolerated by hMSCs (van Hengel et al., 2017; Razzi et al., 2020) suggesting a higher sensitivity of human macrophages to Ag than hMSCs. These findings are also consistent with the previous studies reporting no cytotoxic effects of AgNPs on hMSCs when directly applied on top of the cells at low concentrations (≤0.01 g/L) and no impairment of the osteogenic differentiation of hMSCs (Pauksch et al., 2014; Sengstock et al., 2014). The toxicity of AgNPs against cells is related to two main mechanisms which could be involved in the results obtained: i) contact-killing in which the membrane of the cells coming into contact with surfaces containing AgNPs is damaged and ii) Ag ion-mediated killing in which the ions leached from AgNPs cause cell death (Singh and Ramarao, 2012; Akter et al., 2018). Our findings also indicate that the presence of AgNPs might somewhat compromise the pro-repair behaviour of macrophages as revealed by i) the higher expression of the tissue-repair chemokine *CCL18* by macrophages on the PEO implants than on the PEO + 0.3 Ag implants at both gene and protein levels, and ii) the higher expression of the macrophage surface marker *CD163* on PEO surfaces, characteristic of wound healing (M2a-like) and tissue repair (M2c-like) macrophages (Rees et al., 2015).

When human primary macrophages and hMSCs were co-cultured indirectly, the resulting macrophage response did not considerably differ from the monoculture results. Comparable expression and protein secretion levels of *IL-6* and *CCL18* factors were observed, as well as a slightly more pro-repair phenotype for the macrophages cultured on the PEO surfaces without AgNPs. Although the same factor secretion trend was observed in the co-culture and the monoculture, their levels differ slightly between both cultures. This is most likely due to the differences between the monoculture and co-culture conditions: three times more human macrophages were seeded for the co-culture model and they were cultured 2 extra days comparing to the monocultures with 3 implants per well. Furthermore, hMSCs were observed to osteogenically differentiate, as in the case of monocultures, on both the PEO and PEO + 0.3 Ag surfaces. Previous studies have shown MSCs osteogenesis via the secretion of *OSM* by M1-like macrophages (Guihard et al., 2012). This pro-osteogenic cytokine belongs to the *IL-6* family (Grenier et al., 1999) and was expressed similarly by the cells exposed to both PEO and PEO + 0.3 Ag specimens (Figure 7, B). *PTGS2* is another gene produced by macrophages that is reported to promote the osteogenic differentiation of MSCs (Chen et al., 2021). Pro-inflammatory M1 macrophages were found to enhance the osteogenesis of MSCs and bone formation early in the process via the PTGS2-Prostaglandin E2 pathway (Lu et al., 2017). This gene was also similarly expressed in both implant groups in this study. These results suggest that even though human macrophages showed a slightly lower tissue pro-repair behaviour when cultured with AgNP-incorporated surfaces, the pro-osteogenic response was present in both groups, which is beneficial for osteogenic differentiation of the hMSCs. Previous studies involving the co-culture of these same cells also showed no decrease in osteogenesis in the presence of implants containing Ag ions *in vitro* (Chen et al., 2021). Development of direct co-culture models as well *in vivo* studies would further contribute to understanding the cell-cell communication at the interface with such titanium implants.

The experiments with bacterial cells revealed that the PEO + 0.3 Ag implants could prevent bacterial growth and biofilm formation for both types of the bacteria investigated here. The findings indicated that the direct contact with the surface was toxic to both bacterial strains but the released Ag ions appeared to be more effective against *E. coli* growth as compared to *S. aureus* (Figure 4). These findings are in line with our previous investigations showing that PEO + 0.3 Ag surfaces could significantly reduce the growth of methicillin-resistant *S. aureus* (Necula et al., 2012). In addition, earlier studies have shown a similar faster growth inhibition trend for *E. coli* than for *S. aureus* when exposing bacteria to AgNPs concentrations below 50 mg/mL (Gomaa, 2017). Most importantly, however, the PEO-based surfaces developed in our study prevented the colonisation of the implant surface and the formation of a biofilm while offering a safe dose for human macrophages and hMSCs.

## 5 Conclusion

This study revealed that incorporation of AgNPs by PEO on the surface of biomedical Ti6Al4V implants produced by AM can lead to multifunctional properties, including antibacterial and osteogenic properties, without affecting the viability of human macrophages. The human macrophage response to the optimum silver-incorporated surfaces (*i.e.,* 0.3 g/L), suggested a decrease in their pro-repair tendency. However, this response was shown to not compromise the osteogenic differentiation of hMSCs. Therefore, testing the osteoimmunomodulatory and antibacterial properties of these promising implants in an *in vivo* infection bone model should be performed as the next step towards assessing their potential clinical use.

## Data Availability

The original contributions presented in the study are included in the article/Supplementary Material, further inquiries can be directed to the corresponding authors
