# Supplementary material for: The response of human macrophages to 3D printed titanium antibacterial implants does not affect the osteogenic differentiation of hMSCs
Source: Front Bioeng Biotechnol. 2023 Jun 21;11:1176534. doi: 10.3389/fbioe.2023.1176534 (PMC10319998; doi:10.3389/fbioe.2023.1176534)
Supplement: Supplementary file 1 [file Table1.DOCX]

| **Abbreviation** | **Electrolyte composition** | | |
| --- | --- | --- | --- |
|  | **CaAc (g/L)** | **CaGly (g/L)** | **AgNPs (g/L)** |
| PEO | 24.0 | 4.2 | - |
| PEO + 0.15 Ag | 24.0 | 4.2 | 0.15 |
| PEO + 0.3 Ag | 24.0 | 4.2 | 0.3 |
| PEO + 0.75 Ag | 24.0 | 4.2 | 0.75 |
| PEO + 1.5 Ag | 24.0 | 4.2 | 1.5 |
| PEO + 3.0 Ag | 24.0 | 4.2 | 3 |

**Table S1. List of experimental groups and the corresponding electrolyte composition.**

**Table S2. List of pro- and anti-inflammatory and housekeeper genes with the corresponding primers and probes used for RT-qPCR analysis of human macrophages in monocultures.**

| **Types of gene** | | **Target gene** | **Forward sequence**  **(5’-3’)** | **Reverse sequence**  **(5’-3’)** | **Probe**  **(FAM, 5’-3’)** |
| --- | --- | --- | --- | --- | --- |
| **Pro-inflammatory** | | ***TNF-α*** | GCCGCATCGCCGTCTCCTAC | AGCGCTGAGTCGGTCACCCT |  |
|  |  | ***IL-6*** | TCGAGCCCACCGGGAACGAA | GCAGGGAAGGCAGCAGGCAA |  |
|  |  | ***IL1-β*** | CCCTAAACAGATGAAGTGCTCCTT | GTAGCTGGATGCCGCCAT |  |
| **Anti-inflammatory** | **Cytokines/ chemokines** | ***IL-10*** | CCTGGAGGAGGTGATGCCCCA | GACGCGCCGTAGCCTCAGC |  |
|  |  | ***IL1-RA*** | AACAGAAAGCAGGACAAGCG | CCTTCGTCAGGCATATTGGT |  |
|  |  | ***CCL18*** | GCACCATGGCCCTCTGCTCC | GGGCACTGGGGGCTGGTTTC |  |
|  | **Cell surface markers** | ***CD163*** | GCGGGAGAGTGGAAGTGAAAG | GTTACAAATCACAGAGACCG CT |  |
|  |  | ***MRC1*** | TGGCCGTATGCCGGTCACTGTTA | ACTTGTGAGGTCACCGCCTTC CT |  |
|  | **Growth factors** | ***TGF-β1*** | GTGACAGCAGGGATAACACACTG | CATGAATGGTGGCCAGGTC | ACATCAACGGGTTCACTACCGGC |
| **Housekeepers** | | ***GAPDH*** | CAACGGATTTGGTCGTATTGGG | TGCCATGGGTGGAATCATATTGG | TGCCATGGGTGGAATCATATTGG GGCGCCCCAACCAGCC |
|  |  | ***B2M*** | TGCTCGCGCTACTCTCTCTTT | TCTGCTGGATGACGTGAGTAAAC |  |
|  |  | ***UBC*** | ATTTGGGTCGCGGTTCTTG | TGCCTTGACATTCTCGATGGT |  |

**Table S3. List of early and late osteogenic markers and housekeeper genes with the corresponding primers and probes used for RT-qPCR analysis of hMSCs in mono- and co-cultures.**

|  | **Gene** | **Forward sequence**  **(5’- 3’)** | **Reverse sequence**  **(5’- 3’)** | **Probe**  **(FAM, 5’- 3’)** |
| --- | --- | --- | --- | --- |
| **Early osteogenic marker** | ***COL-1*** | CAGCCGCTTCACCTACAGC | TTTTGTATTCAATCACTGTCTTGCC | CCGGTGTGACTCGTGCAGCCATC |
|  | ***RUNX2*** | ACGTCCCCGTCCATCCA | TGGCAGTGTCATCATCTGAAATG | ACTGGGCTTCTTGCCATCACCGA |
| **Late osteogenic marker** | ***ALPL*** | GACCCTTGACCCCCACAAT | GCTCGTACTGCATGTCCCCT | TGGACTACCTATTGGGTCTCTTCGAGCCA |
|  | ***IBSP*** | TGCCTTGAGCCTGCTTCC | GCAAAATTAAAGCAGTCTTCATTTTG | CTCCAGGACTGCCAGAGGAAGCAATCA |
| **Housekeepers** | ***GAPDH*** | ATGGGGAAGGTGAAGGTCG | TAAAAGCAGCCCTGGTGACC | CGCCCAATACGACCAAATCCGTTGAC |
|  | ***B2M*** | TGCTCGCGCTACTCTCTCTTT | TCTGCTGGATGACGTGAGTAAAC |  |
|  | ***UBC*** | ATTTGGGTCGCGGTTCTTG | TGCCTTGACATTCTCGATGGT |  |

Table S4. List of pro- and anti- inflammatory, tissue-repair, pro-osteogenic and housekeeper genes with the corresponding primers and probes used for RT-qPCR analysis of macrophages in the co-culture model.

|  | **Gene** | **Forward**  **(5’- 3’)** | **Reverse**  **(5’- 3’)** | **Probe**  **(FAM, 5’- 3’)** |
| --- | --- | --- | --- | --- |
| **Pro-inflammatory cytokine** | ***IL-6*** | TCGAGCCCACCGGGAACGAA | GCAGGGAAGGCAGCAGGCAA |  |
| **Tissue-repair chemokine** | ***CCL18*** | GCACCATGGCCCTCTGCTCC | GGGCACTGGGGGCTGGTTTC |  |
| **Anti-inflammatory surface marker** | ***CD163*** | GCGGGAGAGTGGAAGTGAAAG | GTTACAAATCACAGAGACCGCT |  |
| **Pro-osteogenic** | ***OSM*** | GACGCTGCTCAGTCTGGTCC | AGCACGCGGTACTCTTTCG |  |
|  | ***PTGS2*** | TaqMan assay on demand: Hs00153133_m1 (Applied Biosystems, Foster city, CA, USA) | | |
| **Housekeepers** | ***GAPDH*** | ATGGGGAAGGTGAAGGTCG | TAAAAGCAGCCCTGGTGACC | CGCCCAATACGACCAAATCCGTTGAC |
|  | ***B2M*** | TGCTCGCGCTACTCTCTCTTT | TCTGCTGGATGACGTGAGTAAAC |  |
|  | ***UBC*** | ATTTGGGTCGCGGTTCTTG | TGCCTTGACATTCTCGATGGT |  |


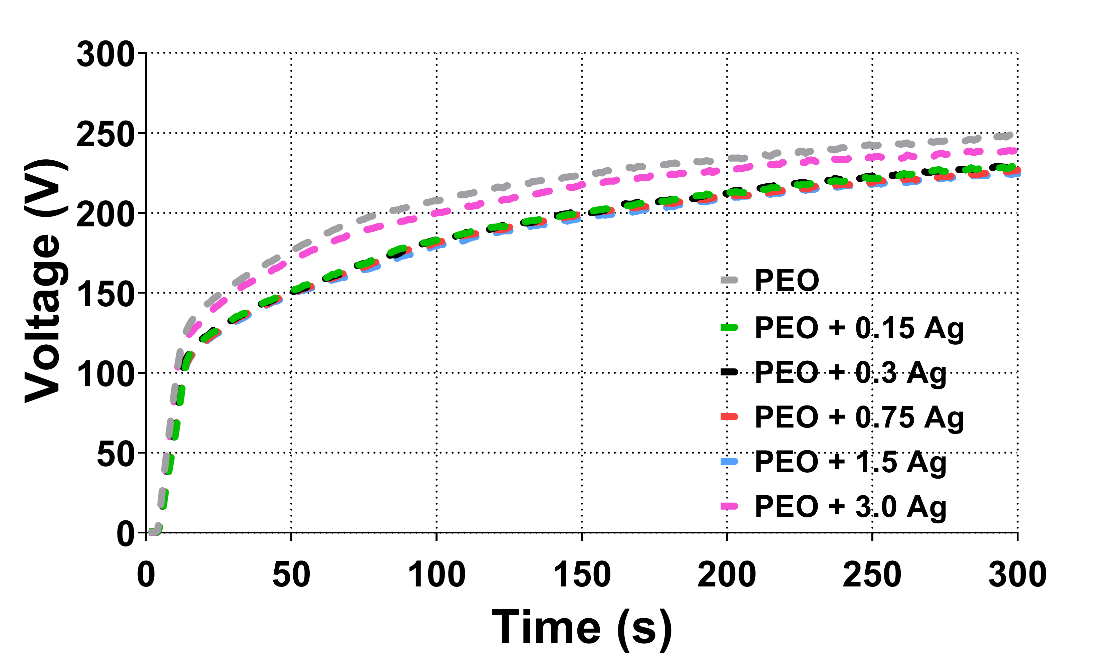


Figure S1. Representative voltage transients recorded during PEO biofunctionalisation.
